# A Model for *Sclerotinia sclerotiorum* Infection and Disease Development in Lettuce, Based on the Effects of Temperature, Relative Humidity and Ascospore Density

**DOI:** 10.1371/journal.pone.0094049

**Published:** 2014-04-15

**Authors:** John P. Clarkson, Laura Fawcett, Steven G. Anthony, Caroline Young

**Affiliations:** 1 Warwick Crop Centre, School of Life Sciences, University of Warwick, Wellesbourne, Warwick, United Kingdom; 2 ADAS UK Ltd, Pendeford House, Pendeford, Wolverhampton, United Kingdom; 3 ADAS UK Ltd, ADAS Drayton, Defra Drayton, Stratford upon Avon, United Kingdom; Georg-August-University of Göttingen Institute of Microbiology & Genetics, Germany

## Abstract

The plant pathogen *Sclerotinia sclerotiorum* can cause serious losses on lettuce crops worldwide and as for most other susceptible crops, control relies on the application of fungicides, which target airborne ascospores. However, the efficacy of this approach depends on accurate timing of these sprays, which could be improved by an understanding of the environmental conditions that are conducive to infection. A mathematical model for *S. sclerotiorum* infection and disease development on lettuce is presented here for the first time, based on quantifying the effects of temperature, relative humidity (RH) and ascospore density in multiple controlled environment experiments. It was observed that disease can develop on lettuce plants inoculated with dry ascospores in the absence of apparent leaf wetness (required for spore germination). To explain this, the model conceptualises an infection court area containing microsites (in leaf axils and close to the stem base) where conditions are conducive to infection, the size of which is modified by ambient RH. The model indicated that minimum, maximum and optimum temperatures for ascospore germination were 0.0, 29.9 and 21.7°C respectively and that maximum rates of disease development occurred at spore densities >87 spores cm^−2^. Disease development was much more rapid at 80–100% RH at 20°C, compared to 50–70% RH and resulted in a greater proportion of lettuce plants infected. Disease development was also more rapid at 15–27°C compared to 5–10°C (85% RH). The model was validated by a further series of independent controlled environment experiments where both RH and temperature were varied and generally simulated the pattern of disease development well. The implications of the results in terms of Sclerotinia disease forecasting are discussed.

## Introduction

Sclerotinia disease of field grown lettuce caused by the fungus *Sclerotinia sclerotiorum* is a major problem worldwide and in the UK losses of up to 50% have been reported [1]. Infection of lettuce plants is initiated by ascospores which are released from apothecia produced through carpogenic germination of soilborne sclerotia near the soil surface. A lack of resistant lettuce varieties means that Sclerotinia disease control is reliant on fungicides, which target the airborne ascospores, but accurate spray timing to achieve good control and avoid unnecessary applications is difficult and is a challenge in all crops affected by *S. sclerotiorum*. This problem could be addressed by developing forecasting models based on the key biotic and abiotic factors governing the development of Sclerotinia disease in order to try and predict periods of risk. This approach has been attempted for several crops including bean, carrot and oilseed rape [2]–[6]. As inoculum production is one of the key factors governing disease risk, we developed a model to predict germination of *S. sclerotiorum* sclerotia and production of apothecia (and hence ascospore release) in order to identify when infection could potentially begin [7], [8]. However, although several studies have shown direct relationships between numbers of apothecia or airborne ascospores and disease levels in the field [9]–[11], this is not always the case if environmental conditions are not conducive to infection. Hence, an understanding of the conditions required for *S. sclerotiorum* infection and disease development, as well as inoculum production, is required.

Previous work has demonstrated the importance of leaf wetness for *S. sclerotiorum* ascospore germination and infection [12], [13] and a relationship between leaf wetness duration and infection levels has been demonstrated for soybean, white bean and dry bean [9], [14], [15]. In addition, duration of high relative humidity (RH) has also been shown to be an important factor for infection and disease development in aubergine and oilseed rape [16], [17]. However, the effect of temperature, and particularly RH on *S. sclerotiorum* infection and disease development, has not been analysed systematically, nor has this information been implemented in a forecasting model. A wide range of temperatures for infection and disease development has been reported for *S. sclerotiorum*; for instance 10–25°C (optimum 20–25°C) for snap/dry beans with no infection at 5 or 30°C [12], [15], 15–25°C for white bean (optimum 20°C) [14] and 7–26°C (optimum 16–22°C) for oilseed rape [3].

In a previous study, we began to define the conditions required for *S. sclerotiorum* infection and disease development specifically on lettuce, through controlled environment (CE) experiments investigating the effects of temperature, RH and leaf wetness [18]. Ascospore germination on lettuce leaves was optimum at 15–25°C and the rate of Sclerotinia disease development was greatest at 16–27°C [18]. However, we could not establish a relationship between leaf wetness period, or duration of different RH levels (up to 6 d), and disease levels. Although free water or very high RH (≥98%) is required for ascospore germination [12], we found that lettuce plants inoculated with dry ascospores still developed Sclerotinia disease. This led to the hypothesis that for RH<98%, there are still micro sites on lettuce plants in leaf axils and close to the stem base where the RH is high enough for spore germination and infection to occur [18]. This study develops this hypothesis further and describes a model for Sclerotinia disease progress based on the concept of an infection court area centred around the stem base of lettuce plants. The size of the effective infection court area is modified by ambient RH and hence represents the micro sites conducive to infection. The model is derived from mathematical relationships describing the effects of spore density, temperature and humidity on the key stages of spore germination, infection and disease development and determines the proportion of plants in a population that develop Sclerotinia disease. The model was parameterised using data collected in CE experiments investigating the effect of ascospore density, temperature and RH on *S. sclerotiorum* infection and disease development, as well as previously published data quantifying the effect of temperature on spore germination and disease development [18]. The model was validated using a set of independent CE experiments investigating disease development at different temperatures and RH.

## Materials and Methods

### *S. sclerotiorum* Isolate and Production of Ascospores

The isolate of *S. sclerotiorum* (isolate 13; IMI 390053) used in this study was originally obtained from diseased outdoor lettuce in 1996 and has been used in previous research investigating environmental conditions influencing infection and carpogenic germination of sclerotia [7], [8], [18]. Sclerotia were produced as described previously [19] by inoculating sterilised wheat grain with agar plugs of an actively growing culture, and incubating at 20°C for 4–5 weeks. Mature sclerotia were then harvested by wet sieving to select those between 2–5 mm, and dried overnight in an airflow cabinet. To produce apothecia and ascospores, *S. sclerotiorum* sclerotia were buried 1 cm deep in 180 g John Innes No. 1 compost (GEM gardening, Accrington, UK; pasteurised at 110°C for 30 min; maintained at 30% w/w water content) placed in clear plastic boxes (600 ml volume, Malsar Kest Ltd, London, UK) and ‘conditioned’ at 5°C for 40 days. This chilling period ensured that carpogenic germination to produce apothecia would occur after 2–3 weeks subsequent incubation in a CE cabinet at 15°C (12 h light/dark, [7]). Ascospores were collected from mature apothecia by opening the lids of boxes and trapping ejected ascospores onto filter papers as described by Young et al. [18] and stored at 4°C in a desiccator. To produce suspensions of *S. sclerotiorum* ascospores in water, filter papers (4–6) were agitated in 1 L distilled water and the suspension was then filtered through two layers of muslin. Viability was always greater than 90%, as checked by observing germination of ascospores on PDA under the microscope after plating appropriate dilutions of the ascospore suspensions and incubating for 24 h at 20°C.

### Lettuce Plants

Lettuce plants (cv. Calgary) for use in experiments were grown in the glasshouse (min. 15°C) in Levington No 2 compost (Levington Horticulture, Ipswich, UK) in modular trays and subsequently transplanted into 9 cm plastic pots (Levington M2 compost) with watering from below. In all experiments, plants were inoculated with *S. sclerotiorum* ascospores when they were 4–6 weeks old with 5–7 fully expanded leaves.

### Model Concept, Structure and Functions

The *S. sclerotiorum* infection model for lettuce was developed to calculate the rate of disease development in a population of lettuce plants (i.e. the number of plants showing symptoms over time), and also the final percentage of diseased plants. The model assumes that all successful infection events result in visible disease symptoms i.e. wilting and collapse of the plant, and that plants are exposed on only one occasion to ascospore inoculum.

In the model, the process of disease development is conceptually divided into three phases: germination, infection, and disease expression, which are influenced by spore density, temperature and RH. The model functions related to these phases were parameterised using results from CE experiments carried out in this study and also from previously published data [18].

### Model Functions

#### Proportion of diseased lettuce plants in a population

The proportion *P* of a population of lettuce plants which develop Sclerotinia disease can be calculated according to Gregory [20] as:
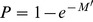
(1)where *M*′ is the average number of effective infection events. An infection event occurs when a spore (or a number of spores) successfully germinates and infects the plant, resulting in visible disease symptoms. This assumes that the total number of infection events is distributed between individual plants according to a Poisson distribution, as observed by other researchers in relation to Sclerotinia disease incidence [17], [21]. A proportion of plants can be infected several times, possibly resulting in more rapid disease development, while some will escape infection all together.

#### Phases 1 and 2: spore germination and plant infection

The effective number of infection events *M′* is dependent on the effective spore density *SD*’ (spores cm^−2^) on a lettuce plant and the effective area *A*′ (cm^2^) of the infection court where conditions on the plant are suitable for spore germination and infection, such that:
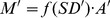
(2)


As stated in the introduction, previous work where lettuce became infected at low RH values despite the requirement of RH ≥98% or free water for ascospore germination, led to the hypothesis that micro sites must exist where RH is close to 100% and conducive to spore germination and infection [18]. The effective infection court area *A*′ in equation 2 therefore represents these conducive micro sites, the total area of which is modified by ambient RH. Hence at low RH, a large proportion of spores will be in areas of the plant where conditions are not suitable for germination or infection. The effective infection court area *A*′ can therefore be represented as a function of ambient relative humidity *RH* (%):
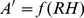
(3)


The effective spore density *SD*′ is a function of the proportion *G* of the true spore density *SD* on the plant that can successfully germinate in the infection court area, such that:
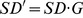
(4)


The density dependence effect implies that at high concentrations, the spores will compete between themselves for the available microclimate sites. The value of the *G* parameter is determined by temperature. The effective number of infection events *M′* is therefore dependent on RH which modifies *A*′, and temperature, which modifies *SD’*. The actual infection court area is not known; therefore, the model is parameterised to represent only the relative change in the court area with RH.

#### Phase 3: disease development

The proportion of infected lettuce plants that show visible symptoms *DP* within a given time duration *L* (days) following infection can be described statistically by a generalised Michaelis-Menten function [22]:
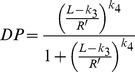
(5)where *DP* is the proportion of plants showing visible disease symptoms, *k_3_* is a response lag (days), *k_4_* is a shape parameter, and *R*′ (days) is the time to the appearance of disease symptoms. The parameters *k_3_* and *k_4_* are constant, but the parameter *R*′ is a function of both temperature *T* (°C) and the average number of effective infection events *M′*:


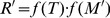
(6)

### Parameterisation of Model Functions

In order to estimate the number of effective infections *M′* (equation 1), and hence the proportion of lettuce plants with disease symptoms *DP* (equation 5), the model functions related to the three conceptual phases of spore germination, plant infection and disease expression were parameterised. This was done using data from previous CE experiments investigating the effect of temperature on spore germination and disease development [18], as well as data from new experiments investigating the effect of spore density and RH on disease development. Once assembled, the model was validated using three independent CE experiments where both temperature and RH were varied.

#### Phase 1: Spore germination, effect of temperature

The effect of temperature on *S. sclerotiorum* ascospore germination was quantified previously on lettuce leaves (cv. Calgary) by inoculating plants with a suspension of ascospores (5×10^4^ ml^−1^), and maintaining continuous leaf wetness over a 28 h period at a range of temperatures between 5 and 30°C [18]. Leaf samples were taken at regular intervals and spore germination was observed under the microscope. Germination started after 2–4 h at optimum temperatures of 15–25°C and reached a maximum after approx. 8–10 h [18]. These data were used to parameterise the proportion of germinated spores on lettuce (*G*), in order to calculate the effective spore density (*SD*′, equation 4).

#### Phase 2: Infection, effect of spore density

Experiments were carried out to determine the effect of *S. sclerotiorum* ascospore density on the proportion of diseased plants (*DP)* hence enabling calculation of the disease development time (days to first symptoms) *R*′, and the effective number of infection events *M′* at each density. Lettuce plants were inoculated with ascospore suspensions at concentrations ranging from 10^1^ to 10^6^ spores ml^−1^. Each plant was sprayed until run-off (approx. 10 ml) using a hand held sprayer and incubated at 20°C for 24 h with continual leaf wetness in a CE cabinet to ensure infection had occurred. In order to relate the concentration of ascospore suspensions sprayed onto lettuce with the resultant ascospore density on leaves, a piece of acetate (1 cm^2^) was placed on each plant at the time of inoculation and removed and dried in an airflow cabinet after the 24 h incubation period. The numbers of spores in three 1 mm^2^ areas per acetate piece were then counted under a microscope allowing calculation of the mean number of spores cm^−2^. Infected lettuce plants were transferred to a greenhouse (mean temperature 20.2°C, mean RH 85%) and disease progress (number of plants with symptoms) recorded at 2–3 day intervals for up to 46 days. A plant was considered diseased when it was completely wilted, and rotting was observed at the stem base accompanied by fluffy white mycelium characteristic of *S. sclerotiorum*. A total of 30 plants were inoculated for each spore concentration treatment (three replicates of 10 plants), and a total of three experiments were carried out. The mean number of leaves per lettuce plant was 5.9, 6.4 and 6.7 for the three experiments, respectively.

#### Phase 3: Disease development, effect of temperature

The disease development time *R*′ (days) that results in disease symptoms is a function of the effective number of infections *M′* but is also a function of temperature (equation 6). In order to quantify the effect of temperature on disease development, data were analysed from experiments carried out previously where lettuce plants were inoculated with spore suspensions at 4×10^5^ spores ml^−1^ and incubated at 20°C with continuous leaf wetness for 24 h to ensure infection. Inoculated lettuce plants were then placed at 8, 11, 16, 22 and 27°C, and 80% RH, and disease development (number of plants with symptoms) recorded at 2–4 day intervals for up to 59 days [18].

#### Phase 3: Disease development, effect of relative humidity

As the effective number of infection events *M′* is a function of effective spore density *SD’* and infection court area *A*′ (equation 2) it is modified both by temperature through *SD’* (equation 4), as well as ambient RH, which we hypothesise determines the size of the effective infection court area *A*′, (equation 3). Experiments were therefore carried out to test the effect of RH on *S. sclerotiorum* infection and disease development. Batches of 21 lettuce plants placed in a cardboard settling chamber (58×40×33 cm) were inoculated from above with dry ascospores released by opening six plastic boxes containing approx. 20 mature apothecia per box. The lid of the cardboard box was then immediately shut, and the spores were allowed to settle for 15 min. Inoculated plants were then placed at 20°C in CE cabinets at 50, 60, 70, 80, 90 or 100% RH, and disease progress was recorded at 2–3 days intervals as before. There were a total of 21 plants per RH treatment and a total of three experiments were carried out. Ascospore density was estimated by placing a piece of acetate on each plant at inoculation and by counting the number of spores as described previously. The mean number of leaves per lettuce plant was 7.1, 6.6 and 5.3 for the three experiments respectively.

### Validating the Sclerotinia Infection Model

In order to validate the infection model parameterisation, Sclerotinia disease development data for three independent CE experiments were collected. Lettuce plants were inoculated with dry ascospores as described earlier and then placed in CE cabinets at 7, 10, 15, 20, 25 or 30°C, and 60, 80 or 100% RH in three separate experiments, respectively. There were a total of 21 lettuce plants per temperature/RH combination (inoculations carried out in replicate groups of 21 plants – seven per temperature/RH combination) and disease development was recorded at 2–3 day intervals for up to 117 days as before. In each set of experiments a ‘standard’ treatment where lettuce plants were kept at 20°C and 100% RH was always included. In these experiments, the lettuce plants were slightly smaller than used previously and the mean number of leaves per lettuce plant was 4.5, 5.4 and 4.4 in each of the three experiments, respectively. Model fit to the observed disease development data was assessed by calculating the root mean square error (RMSE) given by:
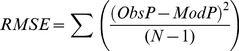
(7)where *ObsP* = observed proportion of lettuce infected, *ModP* = modelled proportion of lettuce infected and *N* = number of observations.

## Results

### Phase 1: Spore Germination; Effect of Temperature

The effective spore density *SD’* can be related to temperature assuming:
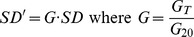
(8)where *SD* is the measured spore density (spores cm^−2^); *G_T_* is the percentage germination at temperature *T* (°C) and *G_20_* is the percentage germination at a reference temperature of 20°C at which spore density experiments were carried out (see next section). The mean rate of spore germination was calculated from the data collected by Young et al., [18] (Table 1) under conditions of continuous leaf wetness and fitted using the temperature response function of Yan and Hunt [23]. The percentage rate of germination (h^−1^) was calculated as:

**Table 1:** Effect of temperature on percentage and rate of germination of *S*. *sclerotiorum* ascospores on lettuce leaves after 10 h (after Young et al. [18]).

Temperature (°C)	Mean % ascospores germinated	Rate of ascospore germination (% hr^−1^)
5	0.22	0.03
10	12.1	1.51
15	38.6	4.82
20	46.8	5.86
25	43.0	5.38
30	0.0	0.00



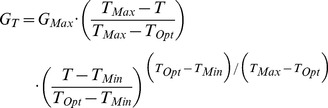
(9)This resulted in a maximum rate of ascospore germination (*G_Max_*) of 6.2% h^−1^. The optimum temperature (*T_Opt_*) was 21.7°C, and the minimum (*T_Min_*) and maximum (*T_Max_*) temperatures were equal to 0.0°C and 29.9°C, respectively (Table 1, Fig. 1A).

**Figure 1:**
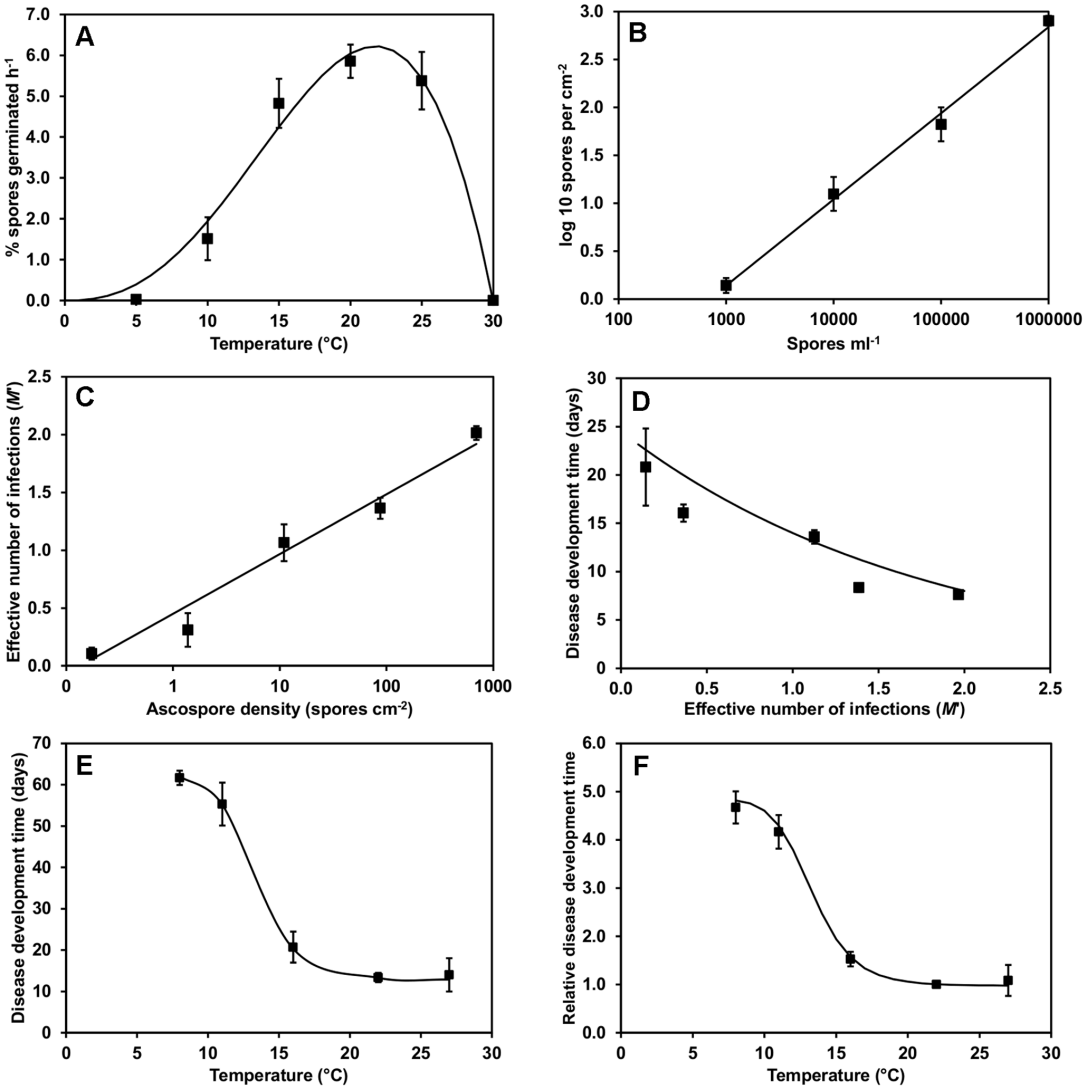
Fitted relationships used in the *S. sclerotiorum* infection and disease development model in lettuce derived for A) temperature and mean rate of *S. sclerotiorum* spore germination h^−1^; B) *S. sclerotiorum* ascospore suspension concentration (spores ml^−1^) applied to lettuce plants and mean final spore density (cm^−2^) measured on leaves; C) mean *S. sclerotiorum* ascospore density (cm^−2^) and effective number of infections; D) effective number of *S. sclerotiorum* infections and mean disease development time (days to first symptoms); E) temperature and mean *S. sclerotiorum* disease development time (days) and F) temperature and relative *S. sclerotiorum* disease development time. Points represent observed data, lines represent fitted functions. Error bars are the standard error of the mean (three replicates).

### Phase 2: Infection, Effect of Spore Density

From the experiments where different *S. sclerotiorum* ascospore concentrations were applied to lettuce plants, a relationship was first derived between leaf ascospore density *SD* (cm^−2^, as measured through microscopy of the acetate pieces placed on leaves), and the spore suspension concentration (spores ml^−1^) applied, (*C*) such that:
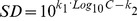
(10)where parameter *k*_1_ was equal to 0.90 and *k*_2_ was equal to 2.56. The regression model explained 99% of the variance (Fig. 1B).

**Figure 2:**
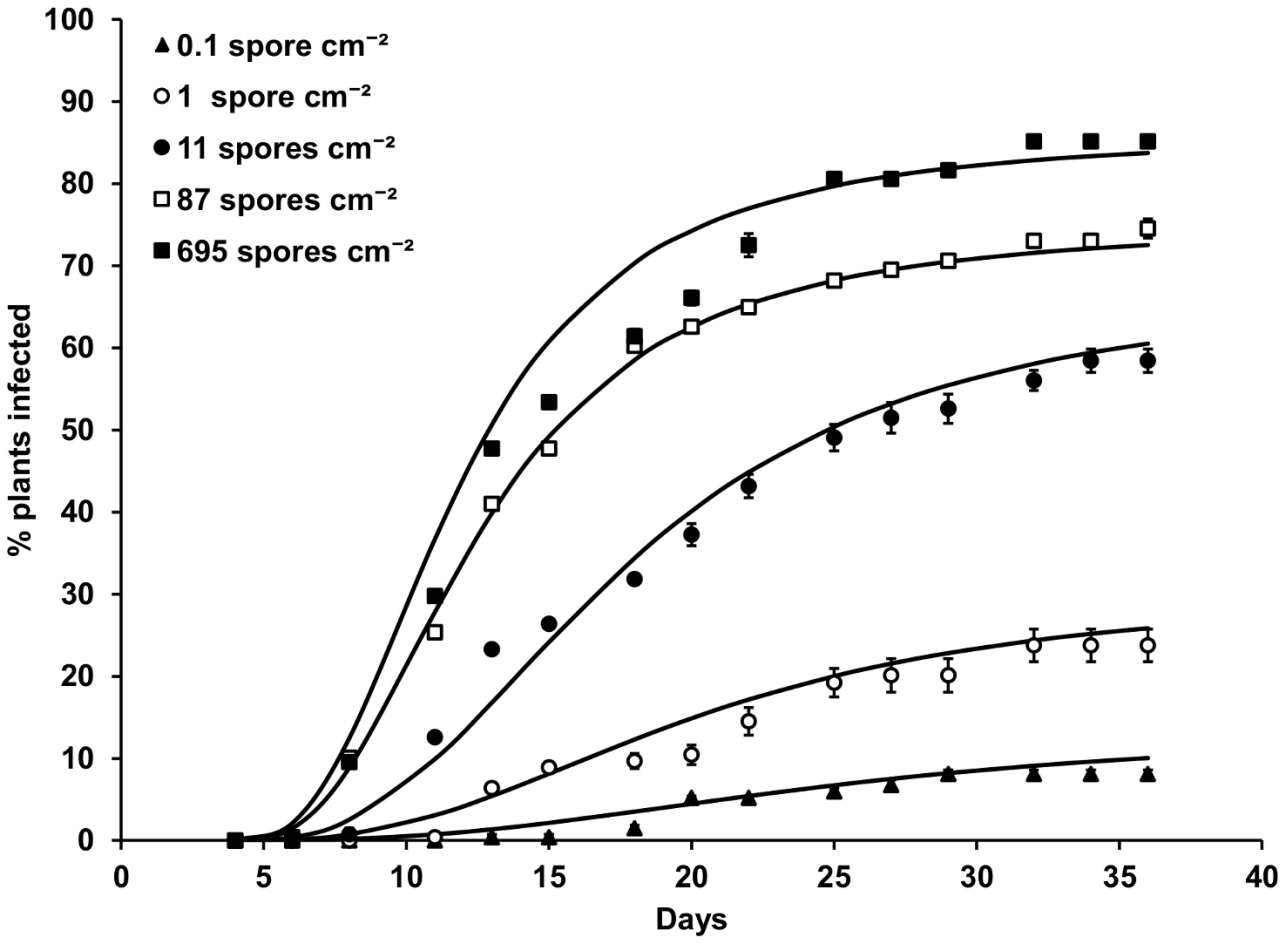
Effect of *S. sclerotiorum* spore density on disease development in lettuce at 20°C and 85% RH. Points represent observed data, lines represent fitted functions. Error bars are the standard error of the mean (three replicates).

The rate of Sclerotinia disease development and final percentage of diseased plants increased with ascospore suspension concentration/density (Fig. 2). Final percentage of diseased plants ranged from less than 20% at 10^2^ spores ml^−1^ (0.1 sp cm^−2^) to more than 80% at 10^6^ spores ml^−1^ (695 spores cm^−2^), while rates of disease development did not increase greatly above 10^5^ spores ml^−1^ (87 spores cm^−2^).

The generalised Michaelis-Menten equation (equation 5) was fitted to these data by iterative optimisation of the sum of squares, using a non-linear conjugate gradient search algorithm. This enabled estimation of the final percentage of diseased plants (*DP*), and hence the apparent number of infections (*M*), and also the time for disease development (*R*′) at each spore density. The lag *k_3_* and shape *k_4_* parameters of the Michaelis-Menten model (independent of spore density) were estimated to be equal to 3.78 and 2.53 days, respectively. A logistic regression relationship (Fig. 1C) between effective spore density *SD’* (spores cm^−2^) and the apparent number of infections *M r*esulting in disease expression, was fitted to the data in the form:
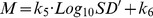
(11)


The coefficient *k_5_* was equal to 0.52 and *k_6_* was equal to 0.45. The regression model explained 97% of the variance.

The time for disease development *R* (days) was also found to decrease with the number of effective infections *M′* (see equation 14) at the reference temperature of 20°C (Fig. 1D), and an exponential regression relationship was fitted of the form:
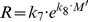
(12)


The coefficient *k_7_* was equal to 24.5 and *k_8_* was equal to −0.56. The regression model explained 87% of the variance.

### Phase 3: Disease Development, Effect of Temperature

The generalised Michaelis-Menten equation (equation 5) was fitted to the Sclerotinia disease development data where infected lettuce plants were incubated at 80% RH at 8, 11, 16, 22 and 27°C [18]. The shape parameter *k_3_* and the response lag *k_4_* constant were held at the previously identified values. As temperature was constant at 20°C for the spore germination/infection phase, and RH was constant for the disease development phase, the maximum percentage disease was also expected to be constant across all experiments. The fitting procedure was used to estimate the effective disease development time *R*′ at each temperature, which was found to decline sharply with temperature between 10 and 15°C (Fig. 1E).

A modified form of the general Michaelis-Menten function was fitted to the data to calculate the effective disease development time *R*′ relative to a reference temperature of 20°C (as used in the spore density experiments):
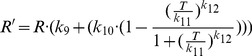
(13)where *R* = time (days) to first visible disease symptoms as a result of infection at the reference temperature of 20°C, the coefficient *k_9_* is equal to 0.97, *k_10_* is equal to 3.87, *k_11_* is equal to 13.32 and *k_12_* is equal to 9.32 (Fig. 1F).

### Phase 3: Disease Development, Effect of Humidity

**Figure 3:**
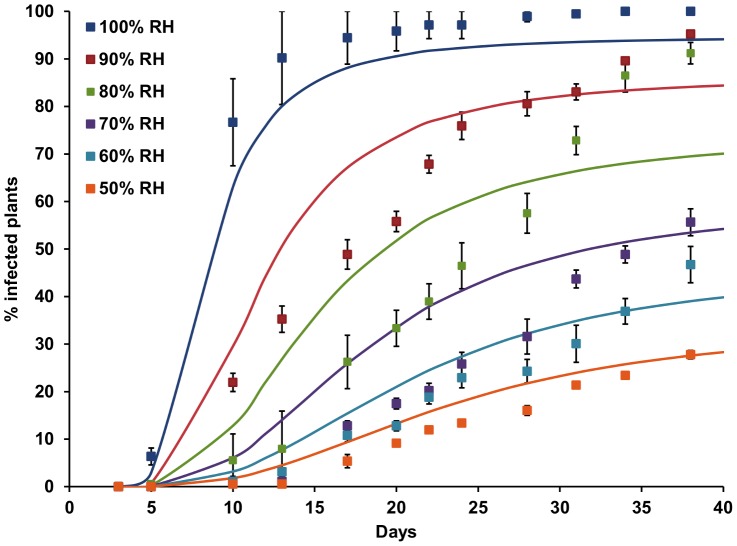
Effect of relative humidity (RH) on *S. sclerotiorum* disease development in lettuce at 20°C. Points represent observed data, lines represent fitted functions. Error bars are the standard error of the mean (three replicates).

**Figure 4:**
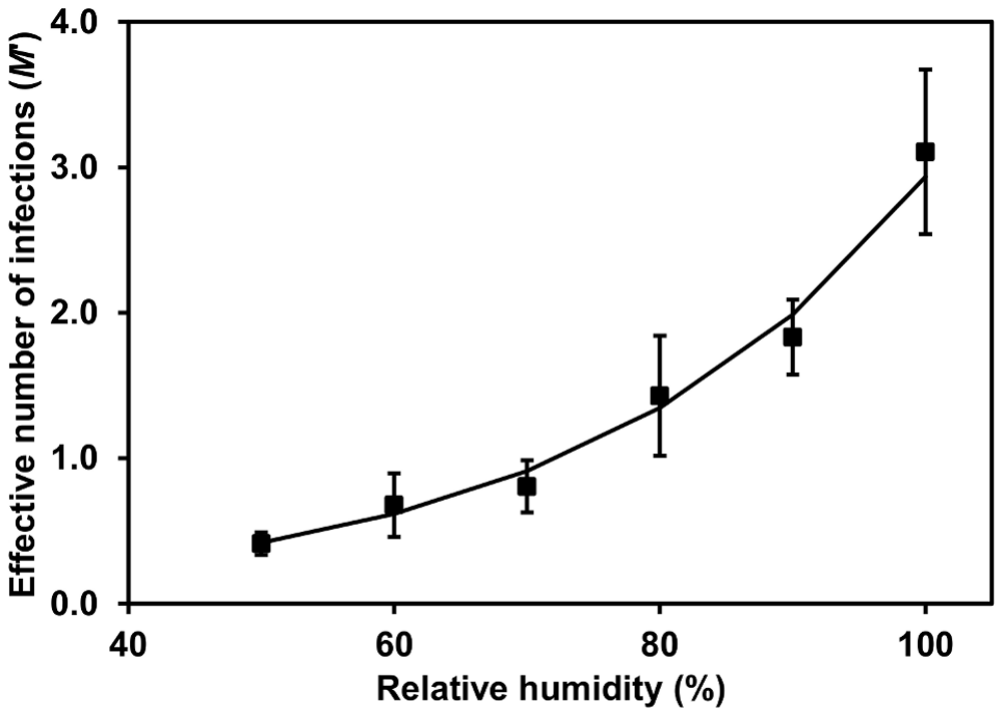
Effect of relative humidity (RH) on effective number of *S. sclerotiorum* infections. Points represent observed data, lines represent fitted functions. Error bars are the standard error of the mean (three replicates).

The mean spore density at inoculation (for batches of 21 plants, ± standard deviation) was 108 (±47), 195 (±93) and 120 (±47) ascospores cm^−2^, respectively, for the three repeat experiments carried out, where lettuce was inoculated with dry *S. sclerotiorum* ascospores and placed at 20°C in CE cabinets at 50, 60, 70, 80, 90 or 100% RH. There were clear effects of the different RH levels on disease development (Fig. 3), with first symptoms appearing most rapidly at 100% RH after 5–7 days over the three experiments. At 80–90% RH, first disease symptoms were observed after 5–17 days. At 50–70% RH, disease progress was slower with first symptoms appearing at 8–21 days and final disease levels of 33–76%. The variation in the response lag (i.e. days to first symptoms) was generally greatest at lower RH levels; e.g. at 50% RH first symptoms appeared between 11 and 21 days over the three experiments. Standard error of the mean time to first symptoms ranged from 0.6 days at 100% RH to 2.9 days at 50% RH. In contrast to the results of previous experiments, there was also little evidence of curvature for some disease progress curves. Nevertheless, the generalised Michaelis-Menten equation (equation 5) was successfully fitted to the data, holding the shape parameter and the relationship between the disease progress rate and the effective number of infection events constant at the previously identified values of *k_3_* and *k_4_*_._ Only the effective number of infection events was allowed to vary and was found to increase significantly with relative humidity. The data were normalised to give the expected number of infections relative to the reference relative humidity of 85% at which the spore density experiments were carried out, and an exponential regression relationship was fitted to the data. The effective number of infections was thus calculated as:
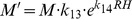
(14)where *RH* is the relative humidity and *M* is the effective number of infections as a function of the effective spore density *SD′*. The coefficient *k_13_* was equal to 0.036 and *k_14_* was equal to 0.039. Plotting *M′* against RH suggested that the number of infection events would be close to zero at RH<40% (Fig. 4). This relationship explained 98% of the variance.

### Validating the *S. sclerotiorum* Infection and Disease Development Model

**Figure 5:**
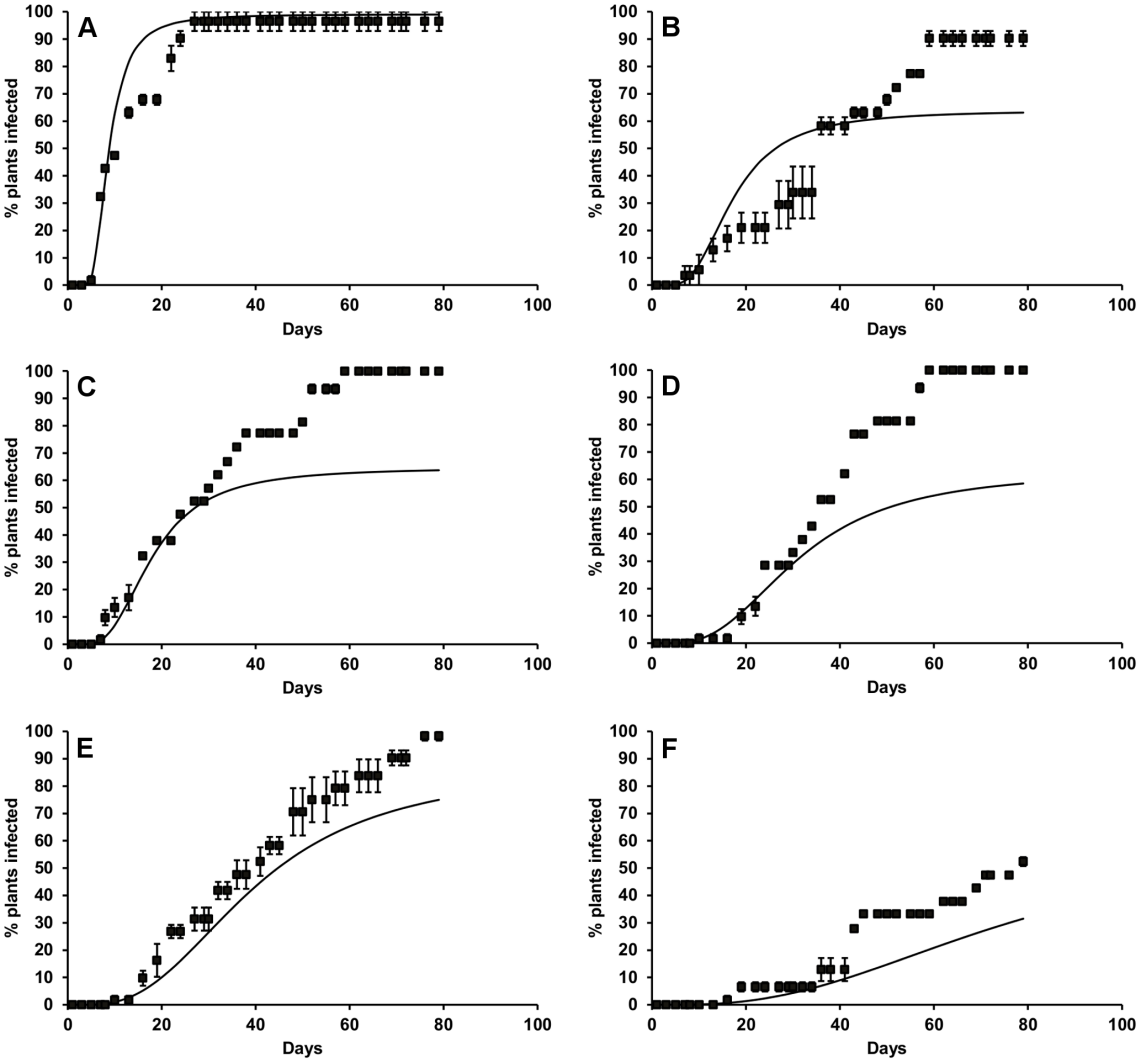
Observed and modelled *S. sclerotiorum* disease development in lettuce at A) 100% RH, 20°C (RMSE = 23.3) and 60% RH at B) 25°C (RMSE = 61.6), C) 20°C (RMSE = 19.7), D) 15°C (RMSE = 100.8), E) 10°C (RMSE = 53.0) and F) 7°C (RMSE = 116.5). Points represent observed data, lines represent model. Error bars are the standard error of the mean (three replicates).

**Figure 6:**
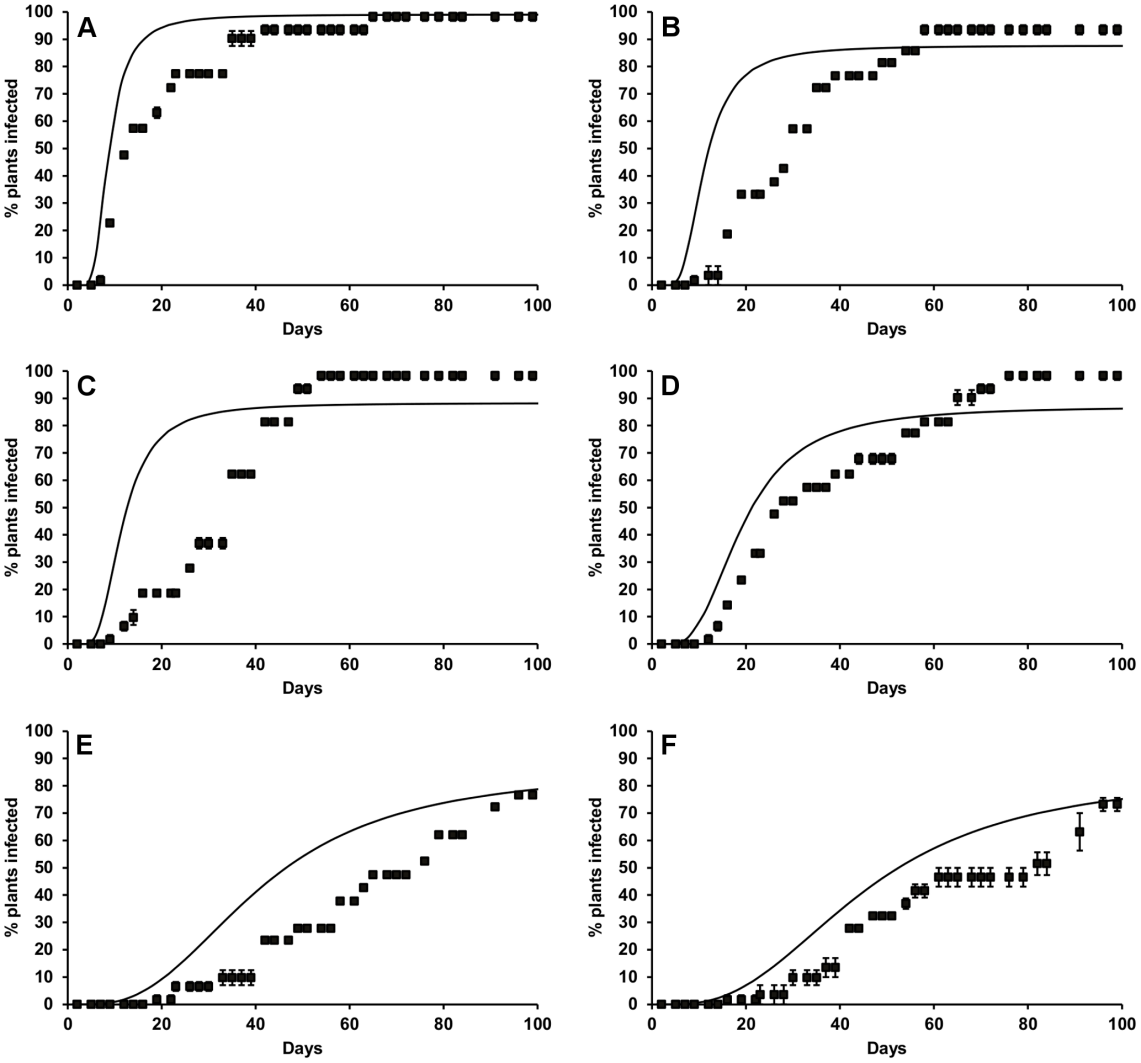
Observed and modelled *S. sclerotiorum* disease development in lettuce at A) 100% RH (RMSE = 51.8), 20°C and 80% RH at B) 25°C (RMSE = 59.4), C) 20°C (RMSE = 54.3), D) 15°C (RMSE = 15.0), E) 10°C (RMSE = 81.8)and F) 7°C (RMSE = 57.5). Points represent observed data, lines represent model. Error bars are the standard error of the mean (three replicates).

**Figure 7:**
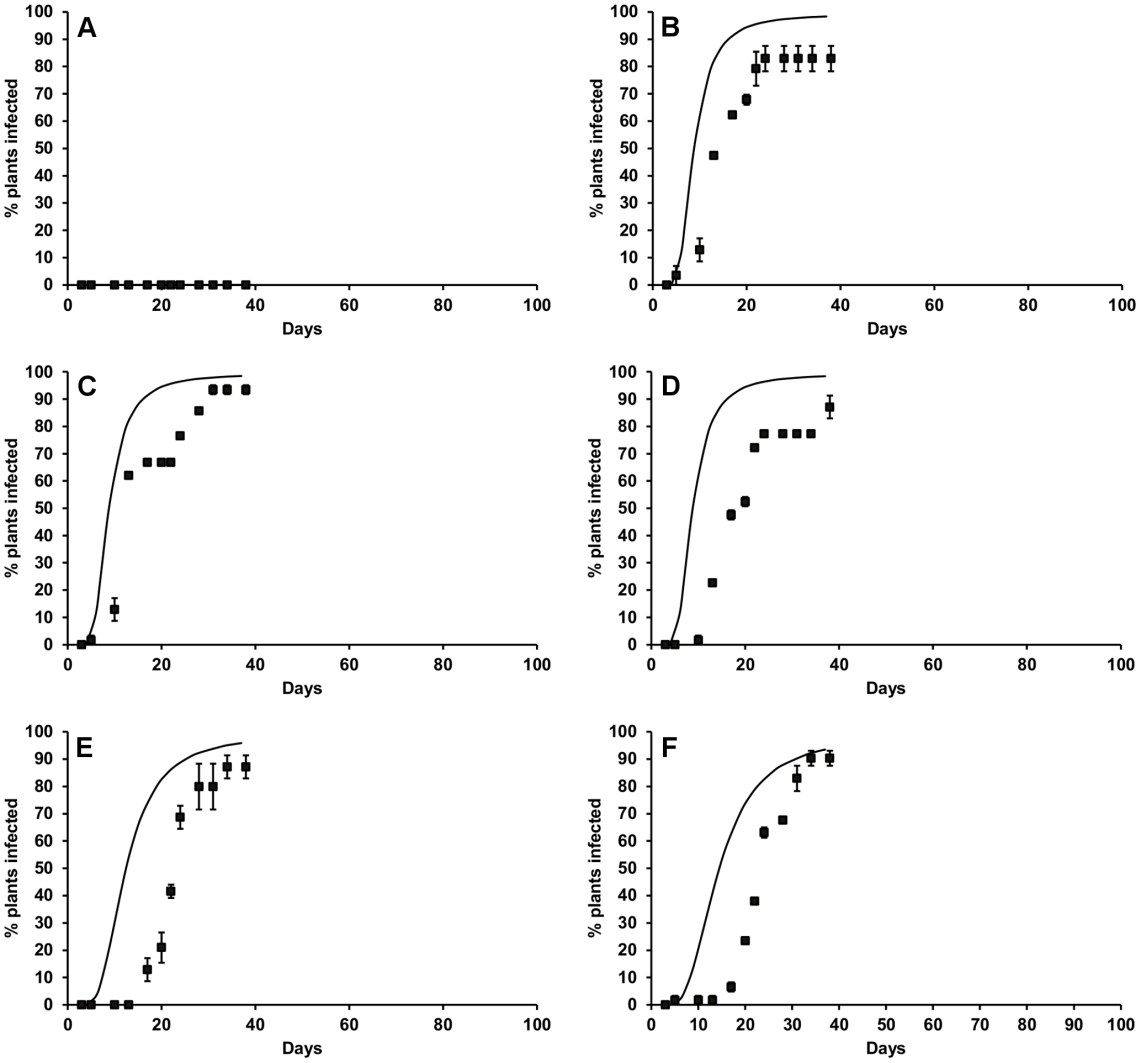
Observed and modelled *S. sclerotiorum* disease development in lettuce at A) 30°C (RMSE = N/A), B) 25°C (RMSE = 37.9), C) 20°C (RMSE = 29.0), D) 15°C (RMSE = 65.4), E) 10°C (RMSE = 48.2) and F) 7°C (RMSE = 27.3). Points represent observed data, lines represent model. Error bars are the standard error of the mean (three replicates).

The mean spore density at inoculation (for batches of 21 plants, ± standard deviation) was 242 (±121), 168 (±97) and 320 (±153) ascospores cm^−2^, respectively, for the three validation experiments carried out at different temperatures at 60, 80 and 100% RH. The ‘standard’ treatment in each of the experiments of 20°C and 100% RH resulted in first symptoms being observed in lettuce plants after 4–8 days over all experiments. For other treatments, there was much wider variation in this observed response lag between replicate batches of plants compared to previous experiments. This variation was smallest at 100% RH (standard error of the mean 0.9–2.4 days over all temperatures) and greatest at 60% RH (standard error of the mean 3.5–8.8 days over all temperatures). Similarly, variation in observed response lag tended to be greater at lower temperatures (standard error of the mean 5.8 days at 20°C and 18.5 days at 7°C over all three humidities). This caused some problems for consistency of the model fit. In addition, the initial model fit to data from the validation experiments indicated significant under-estimation of percentage infection for the treatments at the lower temperatures. However, in the experiments examining the effect of spore density there was a suggestion that the average number of effective infection events was greater for smaller plants. Therefore, the spore density response data for the smaller lettuce from these experiments was used to re-calculate the expected lettuce infection, which resulted in a better model fit overall to the observed data (Figs. 5–7). The model simulated the general pattern of observed disease development adequately, but it tended to underestimate the response lag for all temperatures at 100% RH (Fig. 7) and the final number of lettuce infected at 15, 20 and 25°C at 60% RH (Fig. 5). The total RSME for 7, 10, 15 and 25°C was 351.6, 267.9 and 207.8 for experiments at 60, 80 and 100% RH, respectively, indicating a better model fit at higher humidity. Across all three humidity values the total RSME was 137.7, 191.6, 196.9, 184.1 and 117.1 at 7, 10, 15 and 25°C respectively.

## Discussion

This research demonstrated that spore density, RH and temperature are key factors affecting *S. sclerotiorum* disease development in lettuce using a CE and modelling approach. This is the first time that these factors have been assessed together systematically to produce a simulation model for *S. sclerotiorum* which quantifies the processes of ascospore germination, infection and disease development.

The CE experiments carried out here confirmed our earlier hypothesis that the key infection court for lettuce is at the leaf axils around the stem base, as disease symptoms were always first observed in this area. This was irrespective of whether dry ascospores or water suspensions were used as inoculum and was consistent over a wide range of temperature and humidity. The model concept, that the size of the effective infection court area is mediated primarily by RH, is a novel approach to understanding *S. sclerotiorum* infection and was successful in explaining the experimental observations. The failure to find a relationship between leaf wetness duration and disease incidence in our previous work and the ability of *S. sclerotiorum* ascospores to cause disease at low RH observed in this study suggests that there is always some part of the lettuce stem base where the micro-environment provides conditions necessary for infection. Hence, there is always an effective infection court area, although under low RH (≤50%) conditions, it is very small. The same conclusion regarding the importance of the micro-environment was reached for the lettuce anthracnose pathogen *Microdochium pannattonianum*, where infection also showed minimal dependence on leaf wetness [24]. Lettuce plant architecture and the boundary layer effect makes it very difficult to quantify RH in leaf axils but it is assumed that RH is close to saturation here as this is the requirement for germination of *S. sclerotiorum* ascospores [12]. It is also well documented that a nutrient source is usually required for *S. sclerotiorum* infection as this is a factor governing formation of appressoria [25], [26]. For many crop hosts, nutrients are provided by senescing flower parts and petals as observed for oilseed rape, bean, potato, tomato, broccoli, peas and soybeans [27]–[33]. Very often, these flower parts become lodged in leaf axils where moisture also accumulates, hence promoting stem infections. Nutrients can also be supplied by other means, such as through tissue damage, wounds or senescing leaves, which explains plant infection in the absence of flowering [21], [25], [34]. However, Sedun and Brown [26] also observed infection of sunflower leaves in the absence of flowers, wounds or senescent tissue and this infection was found to be associated with specific sites of sucrose secretion around the junction of the leaf blade and petiole. Given this information from other crops, it is still unclear how the necessary nutrients are provided for infection of young lettuce plants as used in this study. However it was noted in our experiments that even for the relatively small lettuce plants, there was always at least one lower leaf senescing, which was often rapidly colonised by *S. sclerotiorum* mycelium. In the field, ascospores could also be washed down to the lettuce leaf axils by rainfall or irrigation, and any carbohydrates secreted from leaves [35] might also accumulate in this area, both of which would further promote *S. sclerotiorum* infection from the stem base.

This is also the first time that the effect of *S. sclerotiorum* ascospore density on disease development has been specifically examined, and our results showed that disease progression was at a maximum when spore suspensions were applied at 10^5^ spores mL^−1^ or greater, which was equivalent in our inoculation system to 87 spores cm^−2^. There was little disease when spores were applied at 10^2^ spores mL^−1^ which was equivalent to 0.1 spores cm^−2^. Clearly, high spore loads are necessary to ensure that some spores land in the infection court at the stem base. There are no comparable studies where the effect of spore density on Sclerotinia disease has been directly assessed on plants. However, Heran et al., [16] found that lesion size on oilseed rape leaves initiated by petals inoculated with different concentrations of *S. sclerotiorum* ascospores did not increase significantly for spore loads >5×10^2^, which was equivalent to 80 ascospores per petal. Similarly, Herikrishnan and del Rio [36] reported that Sclerotinia disease levels on pinto bean seedlings inoculated with single infected flowers increased with ascospore concentrations from 2 to 200 ascospores per flower. The concentration of ascospores in the air has also been related to disease levels in sunflower and oilseed rape [11], [37] and in the latter study, disease incidence did not increase above 150 spores m^−3^.

The CE experiments investigating the effect of RH on Sclerotinia disease development showed that the effective number of infections increased rapidly between 70 and 100% RH, with fewer plants becoming infected at 50 and 60% RH. Hence, as a simple rule based on these data, RH>70% is conducive to rapid disease progression in lettuce. Most previous studies have focussed on establishing the leaf wetness duration periods required for *S. sclerotiorum* infection or lesion development rather than quantifying the effect of RH on Sclerotinia disease. However, Tores and Moreno [17] reported that disease incidence on glasshouse grown aubergine was related to periods >80% RH, while Hannusch & Boland [38] demonstrated that disease could develop on bean at 90% RH, although lower RH levels were not tested. Finally, Koch et al. [3] determined a threshold RH of 80% for stem infection of oilseed rape by *S. sclerotiorum*, as implemented in the forecasting model SkleroPro. From this work, it seems likely that these ambient RH thresholds are largely a surrogate for higher RH/wetness conditions occurring in the relevant infection court areas within different crop canopies.

We have established and quantified clear effects of ascospore density, temperature and RH on *S. sclerotiorum* infection and disease development through the CE studies presented here and previously [18], although modelling and validating these processes is challenging. We validated the model using three independent CE experiments and although the model generally simulated the pattern of disease development at different temperatures and RH quite well, there were still problems under certain conditions related to underestimation by the model of the final number of lettuce infected at lower RH (60%), and a shorter lag phase compared to the observed data at higher RH (80, 100%). It was clear that even in CE, sub-optimal conditions (low temperature or low RH) for infection and disease development resulted in considerable variability in disease progression curves between replicate batches of lettuce plants in some cases. This may well be due to the difficulty in controlling the ascospore density applied to plants in our experiments. As the effect of RH in the absence of any leaf wetness was being tested, this necessitated the use of dry ascospores for inoculation (as would occur in the natural environment). This involved uniform production of apothecia in closed containers which is difficult and time-consuming to do and relies on ‘puffing’ of spores following opening of the container lids. The number of spores released in this system and the uniformity of their dispersal amongst the lettuce leaves and plants is hence not easily controlled, and individual lettuce plants therefore had a range of spore densities, as shown by the observed variation in mean spore density between and within experiments measured on acetate pieces placed on leaves. This leads to variation in spore density in the infection court area, especially when this area is conceptually small, such as under low RH conditions. Given the importance of spore density in the infection court area in determining the number of effective infections, this is probably the major factor resulting in the observed variation in disease development between repeat experiments and the consequent problems with the model fit. This situation is difficult to resolve but could be improved by carrying out additional replicated experiments with a larger number of plants. However, this approach would require large numbers of apothecia to be produced, which is challenging as evidenced by the lack of any other *S. sclerotiorum* infection studies that have used dry ascospore inoculum.

The importance of *S. sclerotiorum* ascospore density in relation to disease development on lettuce also presents problems in terms of using a simulation model, as presented here, as a means of predicting Sclerotinia disease in the field. It is likely that there will be an additional requirement to measure ascospore density for accurate disease prediction. As Sclerotinia disease development reaches a maximum at a given ascospore density it may be possible to establish threshold values below which the numbers of infected plants becomes insignificant. This could be done using spore trapping and quantitative PCR as developed by Rogers et al., [39]. These researchers demonstrated that the number of ascospores predicted from qPCR results was related to Sclerotinia disease levels on oilseed rape with a peak of 12 ascospores m^−3^ day^−1^ associated with an epidemic in 2007. Levels of four ascospores m^−3^ day^−1^ resulted in negligible disease levels. Combining quantification and timing of inoculum release with the infection/disease development model driven by recording in-crop temperature and RH may help growers make decisions about scheduling fungicide sprays or implementing other disease management strategies in the future. However, work is required to further validate and develop the model for use in a field situation. This may mean simplifying the model by deriving critical RH and temperature thresholds required for rapid infection and disease development, which would be a more practical approach focussed on predicting the likelihood of infection rather than the actual level of potential crop loss.
